# Differential Regulation of Strand-Specific Transcripts from *Arabidopsis* Centromeric Satellite Repeats

**DOI:** 10.1371/journal.pgen.0010079

**Published:** 2005-12-23

**Authors:** Bruce P May, Zachary B Lippman, Yuda Fang, David L Spector, Robert A Martienssen

**Affiliations:** Cold Spring Harbor Laboratory, Cold Spring Harbor, New York, United States of America; University of Wisconsin, United States of America

## Abstract

Centromeres interact with the spindle apparatus to enable chromosome disjunction and typically contain thousands of tandemly arranged satellite repeats interspersed with retrotransposons. While their role has been obscure, centromeric repeats are epigenetically modified and centromere specification has a strong epigenetic component. In the yeast *Schizosaccharomyces pombe,* long heterochromatic repeats are transcribed and contribute to centromere function via RNA interference (RNAi). In the higher plant *Arabidopsis thaliana,* as in mammalian cells, centromeric satellite repeats are short (180 base pairs), are found in thousands of tandem copies, and are methylated. We have found transcripts from both strands of canonical, bulk *Arabidopsis* repeats. At least one subfamily of 180–base pair repeats is transcribed from only one strand and regulated by RNAi and histone modification. A second subfamily of repeats is also silenced, but silencing is lost on both strands in mutants in the CpG DNA methyltransferase MET1, the histone deacetylase HDA6/SIL1, or the chromatin remodeling ATPase DDM1. This regulation is due to transcription from *Athila2* retrotransposons, which integrate in both orientations relative to the repeats, and differs between strains of *Arabidopsis.* Silencing lost in *met1* or *hda6* is reestablished in backcrosses to wild-type, but silencing lost in RNAi mutants and *ddm1* is not. Twenty-four–nucleotide small interfering RNAs from centromeric repeats are retained in *met1* and *hda6,* but not in *ddm1,* and may have a role in this epigenetic inheritance. Histone H3 lysine-9 dimethylation is associated with both classes of repeats. We propose roles for transcribed repeats in the epigenetic inheritance and evolution of centromeres.

## Synopsis

Centromeres are regions of the chromosome that pull the chromosomes to the correct daughter cell during division. They are surrounded by tens of thousands of short satellite repeats, commonly called “junk” DNA. The authors show that these repeats are transcribed into RNA, which is subject to RNA interference, giving rise to large amounts of small interfering RNA. Transcripts are associated with chromosomes during interphase, and mutants in heterochromatin formation have elevated transcript levels. At least two classes of transcripts are silenced by two different epigenetic mechanisms, in part because of transposons inserted into them. This pattern of insertion and regulation varies between natural accessions of *Arabidopsis.* The authors' results suggest a model for centromere evolution and speciation driven by mismatch between pericentromeric repeats and small interfering RNAs in wide crosses.

## Introduction

**Figure 1:**
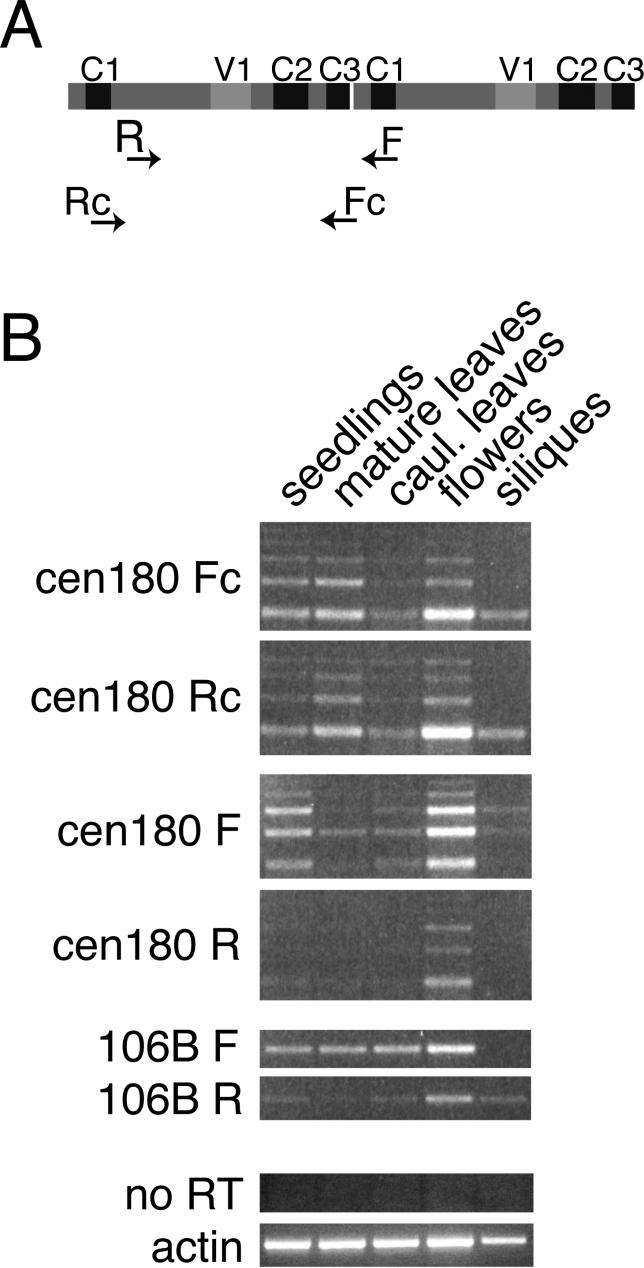
Transcripts from Centromeric Repeats in Landsberg *erecta* (A) cen180 repeats and oligonucleotide primers used in RT-PCR. C1, C2, and C3 are regions of conserved DNA sequence; V is a region of variable sequence. Fc and Rc primers were selected from the conserved regions. (B) RT-PCR of cen180 and 106B repeat transcripts. RT was performed using the primers indicated, followed by PCR with the first primer and a corresponding primer on the other strand. Fc and Rc detect transcripts derived from both strands of bulk repeats, but the F primer detects strand-specific transcripts belonging to a subfamily of these repeats. Primers recognizing *ACTIN2* transcripts served as positive controls. Negative controls lacking reverse transcriptase (“no RT”) tested for DNA contamination.

The centromeric regions of animal and plant chromosomes are large assemblies of thousands of short (approximately 151 to 340 base pairs [bp]) satellite repeats in head-to-tail orientation with interspersed retroelements. In *Arabidopsis thaliana,* these comprise 177- to 179-bp satellite repeats (cen180, also known as pAL1 and AtCon; Figure 1A), *Athila* LTR-retroelements, and 106B repeats, which are 398-bp internal portions of *Athila2* LTRs [1–3]. The only noted common feature among eukaryotic alpha satellites is a binding site for CENP-B, an evolutionary relative of pogo transposase that is necessary for de novo centromere formation [4,5] but not for the centromeres of the human Y chromosome and some marker chromosomes that lack CENP-B [6,7]. In G2 of the cell cycle, a modified histone H3, CENP-A in humans and CenH3 (HTR12) in *Arabidopsis,* is incorporated into centromeric nucleosomes independently of DNA replication [8–10]. A complex of proteins, some of which are directly recruited by CENP-A, then assembles to form the kinetochore that moves the mitotic chromatid or meiotic univalent poleward along depolymerizing microtubules during anaphase.

Although a specific binding site for CENP-A has been hypothesized, constitutive expression of CENP-A results in ectopic deposition at sites throughout the genome [11] and CENP-A has been observed to spread to formerly euchromatic regions lacking repeats during neocentromere creation [12,13]. Transgenic arrays of human centromeric repeats did not attract CENP-A until the cells were treated with an inhibitor of histone deacetylase [14] and yeast Cse4 can functionally substitute for CENP-A in human cells [15]. Thus, deposition of CENP-A may be guided by epigenetic features other than DNA sequence [16,17]. Centromeres appear cytologically as central constrictions flanked by conspicuous pericentromeric heterochromatin. Pericentromeric heterochromatin appears transcriptionally silent: it is depleted of histone H3 methylated at lysine 4 (H3K4me2) as well as histone acetylation, enriched for histone H3 methylated at lysine 9 (H3K9me2), and in mammals, filamentous fungi, and plants, it is enriched for 5-methylcytosine incorporated into the DNA [18–21]. In *Schizosaccharomyces pombe,* which lacks DNA methylation, the central region mediates attachment to the kinetochore while the heterochromatic outer regions attract H3K9me2, Swi6, and cohesin [22]. Unexpectedly, the heterochromatic repeats are transcribed and subject to RNA interference (RNAi) [23]. RNAi guides histone modification via an interaction between complexes containing small interfering RNAs (siRNAs) and nascent transcripts at the locus [24–26]. H3K9me2 in turn binds a repressor, Swi6 in yeast and heterochromatic protein 1 (HP1) in plants and animals, that propagates a more densely coiled, transcriptionally silenced chromatin structure [22]. In fission yeast, Swi6 interacts with the cohesin complex [27] so that the outer regions remain associated longer during mitosis than the central region or the chromosome arms [28]. Lagging chromosomes are observed at high frequency (20%) in mutants defective in heterochromatin formation such as *clr4* (H3K9me2 methyltransferase), *swi6* (HP1 homolog), *rad21/cohesin,* and RNAi [24,29].

This connection between RNAi and centromeric silencing has since been extended to mammalian, insect, and avian cells, but it is not known if the interaction with cohesin is common to other organisms [30–32]. *Drosophila* centromeres have subregions containing either the centromeric histone (Cid) or H3K4me2, and these cluster into higher order domains, with Cid clusters facing the kinetochore [33]. Mutants in *Arabidopsis* homologs of *swi6* and *rad21* have early flowering and meiotic phenotypes, respectively, but lagging mitotic chromosomes have not been reported [34,35].

DNA methylation is found in many eukaryotes (although not extensively, if at all, in *S. pombe, Drosophila,* or *Caenorhabditis elegans*) and interacts with histone deacetylation and H3K9me2, but the order of steps is unclear: In *Arabidopsis,* loss of H3K9me2 is accompanied by loss of DNA methylation [36–39] and loss of DNA methylation is accompanied by loss of H3K9me2 [39–41], although the latter effect may not be direct [20]. The DNA methyltransferase *CHROMOMETHYLASE3 (CMT3),* the histone H3 lysine-9 methyltransferase *KRYPTONITE (KYP),* and the RNAi component AGO4 maintain hypermethylation induced by transcription of long inverted repeats [37,42–45]. In contrast, *DNA METHYLTRANSFERASE1 (MET1)* and *HISTONE DEACETYLASE6 (HDA6)* are required to maintain silencing of promoters induced by transcription of short inverted repeats [46]. Despite the presence of siRNA, other components of the siRNA pathway have not been recovered in these screens, and AGO4 is not required for silencing mediated in *trans* by short inverted repeats [45]. But AGO4 is required, along with *DICER-LIKE3 (DCL3)* and *RNA-DEPENDENT RNA POLYMERASE2 (RDR2),* for *DRM1-* and *DRM2*-dependent DNA methylation and silencing of transgenes when they are introduced via agrobacterium-mediated transformation [47]. Similarly, two pathways have been identified for transposon silencing in *Arabidopsis:* One involves *CMT3, KYP,* and *AGO1,* and the other involves *MET1, DDM1,* and *HDA6* [39]. In this second pathway, resilencing of some but not other transposons in backcrosses suggests a role for siRNA in the establishment of silencing [39]. For most transposons, a role for siRNA in the maintenance of silencing is not apparent in *dcl3* mutants, although other dicers can compensate for the loss of *DCL3* [48] so that such a role cannot be ruled out.

We have found that centromeric satellite repeats in *Arabidopsis* are transcribed strand-specifically and that the transcripts remain associated in the nucleus. Only a subset of 180-bp repeats is transcribed, but the repeats are partially silenced by an RNAi-based system including *DCL1, AGO1, CMT3, KYP,* and *HDA6/SIL1*. Loss of silencing is inherited epigenetically in backcrosses. Other 180-bp repeats are silent in wild-type (WT) but transcribed in *met1, ddm1,* and *hda6*. *Athila2* LTR retroelements direct the transcription of these satellite repeats, and silencing is restored in *met1* and *hda6* backcrosses. Consistent with differing patterns of retroelement insertion, different repeats are transcribed in the Landsberg and Columbia strains of *Arabidopsis,* providing a possible mechanism for the rapid evolution of centromeric satellite repeats.

## Results

### Centromeric Repeats Are Transcribed and Remain in the Nucleus

Arabidopsis cen180 repeat families are 78% to 96% identical in sequence. Variant repeats are located on different chromosomes [49] and may, like human alpha satellites, form subdomains [50], but each repeat contains common conserved regions [2]. We designed cen180 Fc and Rc primers (Figure 1A) to recognize two conserved regions, and these primers amplified a large number of repeats from genomic DNA. Transcripts from centromeric repeats were detected by RT followed by PCR. “F-strand transcripts” are transcribed from the forward (Watson) DNA strand and reverse transcribed into cDNA using an F primer; “R-strand transcripts” are transcribed from the reverse (Crick) DNA strand and reverse transcribed into cDNA using an R primer. Using the highly conserved Fc and Rc primers, transcripts from both strands could be detected (Figure 1B). However, transcripts from each strand could be derived from different subfamilies of repeats. In order to test this possibility, specific primers were used (F primer and R primer, Figure 1A) to amplify only a subset of cen180 repeats [20]. In this case, we found that F-strand transcripts were much more abundant than R-strand transcripts in vegetative and floral tissues, suggesting strand-specific transcription of individual subfamilies of repeats (Figure 1B). Transcription from only one strand was consistent with transcripts initiating in tandem orientation from the repeats, while the amplification of 180-bp multimers indicated that transcripts did not terminate within each repeat. Transcript levels were highest in young seedlings and developing inflorescences, indicating expression in dividing cells.

**Figure 2:**
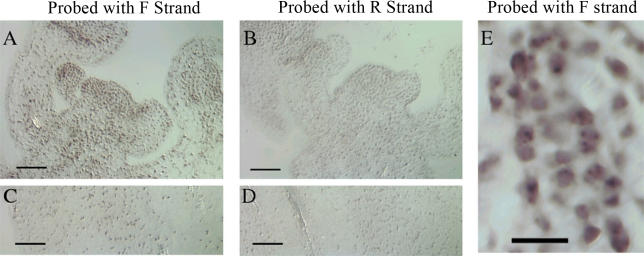
In Situ Hybridization of Centromeric Probes with Longitudinal Sections of Inflorescences (A) Inflorescence meristem and developing buds probed with digoxigenin-labeled F strand of cen180 F + R PCR products and (B) probed with R strand. Mature tissue about 5 mm below the meristem (C) probed with F strand of cen180 and (D) probed with R strand. The scale bars are 50 μm. (E) More highly magnified view of nuclei from developing buds probed with F strand (scale bar is 10 μm).

The same subfamily of cen180 repeats were amplified from genomic DNA and used as strand-specific probes for in situ hybridization (see Materials and Methods). In longitudinal sections of WT inflorescences, F-strand transcripts were more abundant than R-strand transcripts (Figure 2), consistent with RT-PCR (Figure 1B). Young, proliferating tissues (Figure 2A and 2B) had more signal than mature tissue (Figure 2C and 2D). Strand and tissue specificity indicated that the signal came from cellular RNA and was not due to background hybridization with fortuitously denatured DNA. Under higher magnification, small punctate nuclear signals were seen with the F-strand probe, consistent with nascent transcripts remaining at the centromere (Figure 2E). In contrast, what little signal could be detected with the R-strand probe was nuclear but not punctate (Figure 2B and 2D).

### Satellite Repeat Transcripts Are Regulated by DNA Methylation, Histone Modification, and RNAi

**Figure 3:**
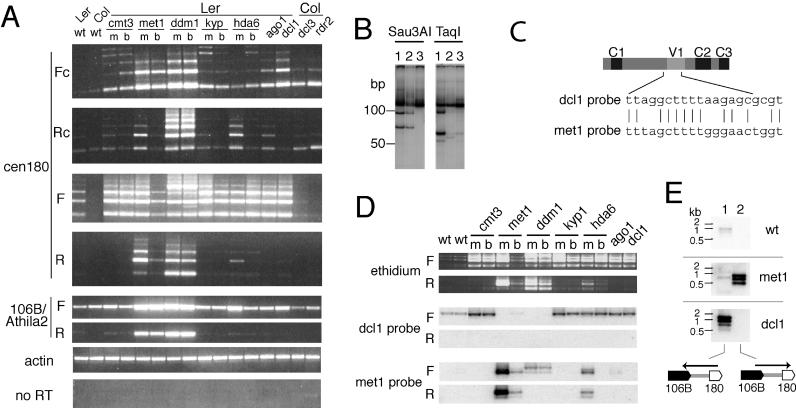
Regulation of Centromeric Transcripts (A) Strand-specific RT-PCR of cen180 repeats. RT was performed with forward and reverse primers indicated to the left of each panel. Mutants in DNA methylation *(cmt3, met1),* chromatin remodeling *(ddm1),* histone modification *(kyp, hda6),* and RNAi *(dcl1, ago1)* were in the Landsberg *erecta* (Ler) ecotype. *rdr2* and *dcl3* were in the Columbia background. m indicates homozygous mutants and b indicates backcrosses of mutants to WT (wt). Positive controls were primers recognizing the *ACTIN2* gene; negative controls lacked reverse transcriptase (no RT). (B) Restriction site analysis of repeats. Repeats were amplified by cen180 F+R primers from genomic DNA (lanes 1), cDNA from *met1* mutants (lanes 2), and cDNA from *dcl1* mutants (lanes 3). Repeats in genomic DNA that have *TaqI* sites are not transcribed in either mutant, while those with Sau3AI sites are not transcribed in *dcl1*. (C) Oligonucleotide probes specific for the subfamilies of F + R cen180 repeats expressed in *dcl1* and *met1* mutants. (D) Strand-specific RT-PCR of cen180 repeats. RT was performed with F or R primers as in (A). Southern hybridization of these products with specific oligonucleotide probes distinguished subfamilies of repeats that were differentially regulated. (E) Detection of read-through transcripts between cen180 and 106B repeats by strand-specific RT-PCR. 106B Fout (lane 1) and cen180 F (lane 2) primers were used to prime cDNA, followed by PCR amplification. The configuration of the transcripts is shown below each lane. Other combinations of primers did not give detectable products. RNA was prepared from aerial tissues from 28-day-old plants.

In *S. pombe,* H3K9me2 depends on RNA silencing of pericentromeric repeats [23], and vice versa [51]. Similarly, in *Arabidopsis,* H3K9me2, RNAi, and DNA methylations are mutually interdependent [39,45]. We examined the level of pericentromeric transcripts in *Arabidopsis* mutants defective in DNA and histone methylation as well as in RNAi (Figure 3A). Elevated transcript levels were detected in *dcl1–9* (a weak allele of *DICER-LIKE1*), *ago1–9* (a strong allele of *ARGONAUTE1*), *rdr2–1* (a T-DNA insertion in *RNA DEPENDENT RNA POLYMERASE2*), *dcl3–1* (a T-DNA insertion in *DICER-LIKE3*), *kyp-2* (a splice-site mutation in histone H3 K9 methyltransferase), *hda6/sil1* (an allele of *HISTONE DEACETYLASE6*), *cmt3-m5662* (a null allele of the CNG DNA methyltransferase), *met1–1* (a strong allele of the CG DNA methyltransferase), and *ddm1–2* (a hypomorphic allele of the swi/snf chromatin remodeling ATPase). Strand-specific RT-PCR (Figure 3A) indicated that F-strand transcripts were elevated in abundance in *cmt3, kyp, hda6,* and the RNAi mutants, whereas both F- and R-strand transcripts accumulated in *met1* and *ddm1.* In backcrosses to WT, elevated transcript levels were inherited epigenetically from *cmt3, ddm1,* and *kyp.* Reestablishment of silencing was observed for both strands in *met1*/+ backcrosses and on one strand in *hda6*/+ backcrosses. *dcl1–9* and *ago1–9* were not tested in this way due to sterility [39]. Northern analysis of *met1* and *dcl1* mutants revealed heterogeneous transcripts ranging from approximately 0.1 kb to more than 5 kb, indicating multiple, irregular transcription initiation and/or termination sites (Figure S1).

### Satellite Repeat Subfamilies Are Silenced by Different Mechanisms

One explanation for this differential regulation was that further subfamilies of F + R repeats were differentially regulated on each strand. We therefore examined which particular repeats were expressed in *met1* compared with *dcl1, ago1,* and WT using restriction and sequence analysis of cDNA. Digestion of PCR products from cDNA and genomic DNA with TaqI revealed that transcribed repeats, which were sensitive to TaqI digestion, were a minority of those amplified from genomic DNA, most of which were not sensitive (Figure 3B). Further, RT-PCR products from *met1* were digested by Sau3AI, indicating they had Sau3AI sites, but cDNAs from *dcl1* were not digested and must therefore differ in sequence. Transcribed repeats were sequenced and grouped by cDNA sequence similarity using CLUSTALW. Whereas those from WT, *dcl1,* and *ago1* formed mixed clusters, transcripts from *met1* were from a distinct subfamily of repeats (Figure S2).

Diagnostic 20-mers were designed from the most highly diverged satellite cDNAs (Figure 3C) and hybridized to strand-specific RT-PCR products from the various mutants (Figure 3D). Hybridization was exclusive and complementary. F-strand transcripts from the subfamily of repeats expressed in WT were elevated in *cmt3, kyp, hda6, dcl1,* and *ago1,* but transcripts from the R strand were not seen. Elevated transcript levels were inherited epigenetically when *kyp, hda6,* and *cmt3* mutants were backcrossed to WT. The subfamily of repeats expressed in *met1* was transcribed from both strands in *met1* and *hda6* but only from the F strand in *ddm1.* These transcripts could not be detected in WT and were epigenetically inherited in *ddm1/+* but were substantially resilenced in *hda6/+* or *met1/+* backcross progeny*.* In this respect, they resemble *ATGP1* gypsy-class LTR retrotransposons [39,41,52]. Additional R-strand transcripts, detected by RT-PCR in *ddm1,* did not hybridize with either probe, indicating a third subfamily of differentially regulated repeats.

The differentially regulated subfamilies of cen180 repeats are very similar, so sequence differences are unlikely to account for differences in regulation. A more likely explanation was that some cen180 transcripts might be driven by read-through from adjacent retroelements. The 106B LTR-like repeats and *Athila*-class LTR retroelements are integrated throughout pericentromeric regions [53] and are interspersed in random orientation with respect to cen180s (data not shown). F-strand 106B transcripts were detected in WT but were only weakly up-regulated in *cmt3, kyp,* and *hda6* (Figure 3A). Transcripts from both strands were up-regulated in *ddm1* and *met1,* and R-strand transcripts were up-regulated in *hda6*. In this respect, 106B repeats resembled both classes of cen180 repeats, and we examined the possibility they were co-transcribed using cDNA that was reverse transcribed with 106B or cen180 primers (Figure 3E). F-strand co-transcripts originating in cen180 repeat arrays could be detected in WT and *dcl1.* In contrast, reverse strand co-transcripts appeared to originate within 106B and could only be detected in *met1.* Subsequent RACE PCR showed that these co-transcripts originated in the LTR of *Athila2* itself (not shown). Other co-transcripts were not seen. This indicated that differential genetic regulation of each class of cen180 repeat transcript could be explained by differential origin of the transcripts in 106B/*Athila2* and cen180 repeats, respectively.

### Polymorphic Regulation of Repeats

**Figure 4:**
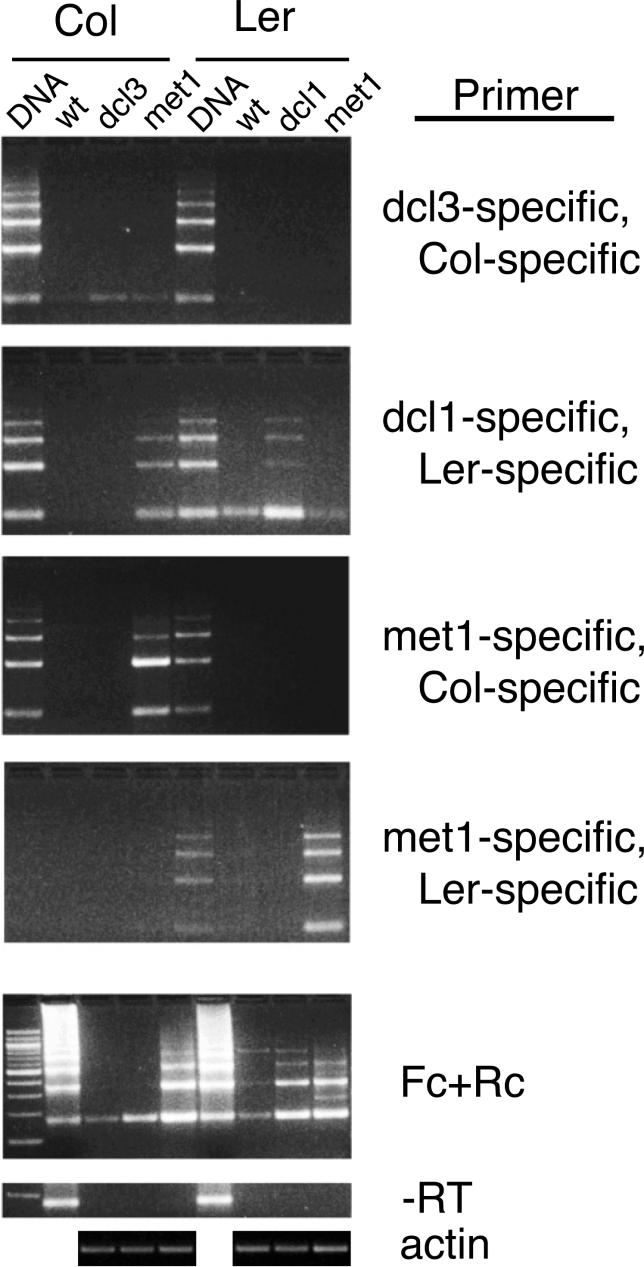
Polymorphic Regulation of cen180 Transcripts Primers were designed to recognize sample cen180 cDNAs from *dcl1* and *met1* mutants of Landsberg *erecta* (see Figure 3C) and *dcl3* and *met1* mutants of Columbia. PCR amplification using genomic DNA templates (DNA) tested for the presence of the particular repeats in the genome of each ecotype. RT-PCRs using RNA detected transcripts in WT (wt) and mutants of each ecotype as indicated. The cen180 Fc + Rc primers served as positive controls in the presence of reverse transcriptase and negative controls in its absence. RNA was prepared from aerial tissues from 28-day-old plants.

Most of the mutants described above were isolated in the Landsberg genetic background, but *rdr2* and *dcl3* were isolated in Columbia, and so this WT strain was also assayed for transcripts. Surprisingly, Columbia and Landsberg transcripts amplified with conserved primers Fc and Rc differed in their regulation (Figures 3A and 4). We therefore sequenced cen180 RT-PCR products from *dcl3* and *met1* mutants of Columbia and designed diagnostic primers for amplification of genomic DNA and cDNA (Figure 4). Primers corresponding to *dcl3*-and *met1*-specific transcripts amplified cDNA from each corresponding mutant in Columbia, as expected. However, no cDNA could be amplified from *met1* or *dcl1* mutants in Landsberg despite the presence of such repeats in the Landsberg genome (indicated by amplification of genomic DNA). Conversely, specific transcripts detected in *dcl1* mutants in Landsberg were found to accumulate in *met1* mutants of Columbia, indicating that the regulation of this class of repeats had changed during the divergence of these two ecotypes. Repeats transcribed specifically in *met1* mutants of Landsberg do not appear to be present in the Columbia genome. The origin of this natural variation is discussed below.

### Histone Modifications Associated with Satellite Repeats

We attempted to examine histone methylation associated with centromeric repeats using semiquantitative chromatin immunoprecipitation, although this proved to be very difficult as previously reported [20,40] because of the very high copy numbers involved. Nonetheless, while quantitative changes were not reliable, qualitative changes could be detected (Figure S3). H3K9me2 was substantially reduced in *kyp* and *ago1* over both classes of repeat, resembling transposons such as *ATCOPIA4* [39] as well as pericentromeric repeats and retrotransposons in *S. pombe* and *Drosophila* [23,31]. In *met1* and *ddm1,* H3K9me2 was largely lost from 106B but only slightly reduced at cen180. H3K4me2 underwent a modest but heritable increase in *ddm1* and was found associated with each class of repeat in WT, perhaps reflecting the transcription of the repeats. The association of H3K4me2 with heterochromatic pericentromeric repeats has also been reported in *S. pombe* [23], while in *Drosophila* [33] it is interspersed with Cid in more central domains.

DNA methylation of centromeric repeats has been extensively investigated by gel blot analysis, and we have obtained similar results (Figure S4). CNG methylation is partial in WT and reduced in *ddm1, cmt3,* and *kyp* [21,37,42,43], but it is unaffected in *hda6* [54]. CG methylation is more extensive than CNG methylation at the centromere in WT and is lost only in *met1* and *ddm1*. However, *met1* affects transcripts initiating in 106B/*Athila2* LTRs and not those initiating within the class of cen180 repeats regulated by RNAi (Figure 3E). *hda6* has a similar effect on 106B transcripts but has no effect on CG methylation, although H3K9me2 is reduced ([54] and data not shown). H3K9me2 was retained in *rdr2* and *dcl3* (data not shown). Thus, neither DNA methylation nor H3K9me2 correlates perfectly with the specific deregulation of 106B/*Athila2*- or cen180-driven satellite repeat transcripts, although complexes responsible for each modification play a major role.

### siRNA from Centromeric Transcripts Depends on *DDM1, DCL3,* and *RDR2*

**Figure 5:**
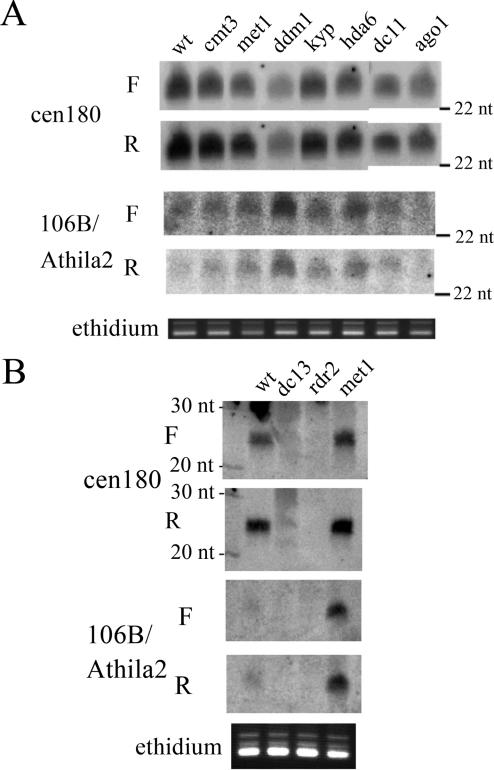
siRNAs from Centromeric Repeats Total RNA was purified from plants of the indicated genotypes, and small RNAs were enriched by solubility in PEG (see Materials and Methods). Mutants and WT (wt) were in the Landsberg *erecta* background (A) or the Columbia background (B). RNA was prepared from aerial tissues from 28-day-old plants.

Centromeric repeat transcripts in *S. pombe* correspond to siRNAs [55] that depend on Dcr1, Rdr1, and Ago1 for their accumulation [56,57]. In *Arabidopsis,* 24-nucleotide siRNAs corresponding to 180-bp centromeric repeats were detected in WT and were unchanged in *met1, hda6, cmt3, ago1, dcl1,* and *kyp* but were reduced in *ddm1* and almost entirely absent from *dcl3* and *rdr2* (Figure 5A and 5B). These blots were deliberately overexposed to show residual levels of 24 nucleotides and smaller classes of siRNA. It is not possible to tell if these are derived from specific subclasses of repeats. Small RNAs corresponding to both strands of 106B repeats were also unchanged in most mutants but were increased in *ddm1,* decreased in *ago1* (Figure 5A), and could not be detected in *dcl3* and *rdr2* (Figure 5B), resembling siRNA derived from the gypsy class retrotransposons *ATGP1* [39] and *Athila2* (data not shown). Both *dcl3* and *rdr2* have been shown to be required for production of endogenous siRNAs, while *dcl1* is required for processing of micro-RNAs [58] and *trans-*acting siRNAs [59,60].

### Lagging Chromosomes Were Not Observed in Mutants Defective in Silencing

In the fission yeast *S. pombe,* mutants defective in centromeric silencing recruit, but fail to retain, cohesin in anaphase pericentromeric heterochromatin, because of the loss of H3K9me2 and associated Swi6 [61]. We therefore examined anaphase in centromeric silencing mutants in *Arabidopsis*. Developing floral or root tissue was fixed in paraformaldehyde and stained with DAPI to detect the presence of lagging or otherwise abnormal chromosomes during anaphase. In WT anaphase cells (N = 20), *rdr2* mutant cells (N = 49), and *ddm1* mutant cells (N = 23), no abnormality was seen (not shown). Further, these mutants were indistinguishable from WT in growth and fertility, unlike mutants in *rad21/cohesin,* which are semisterile due to defects in meiosis [34]. One explanation might be that H3K9me2 was largely retained in *rdr2* and *ddm1,* so that any interaction with Swi6 homologs and cohesin was retained. However, mutants in the H3K9 methyltransferase *kyp* are also fully fertile, and lagging chromosomes were not observed in *kyp* anaphase cells either (N = 24). Differences between the yeast and plant systems are discussed below.

## Discussion

### Silencing of Centromeric Transcripts in Arabidopsis and Fission Yeast

**Figure 6:**
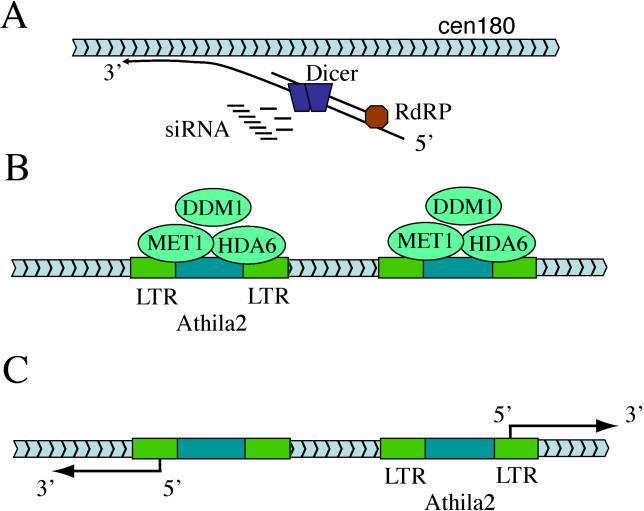
A Model for the Regulation and Evolution of Centromeric Satellite Repeat Transcripts (A) New tandemly arranged repeats are transcribed on one strand and processed by RNAi. (B) Older repeats accumulate retrotransposon insertions that interrupt and silence the transcripts via MET1, HDA6, and DDM1. (C) In *met1, hda6,* or *ddm1* mutants, the silencing complex is lost and promoters from retrotransposon LTRs drive transcription from both strands. cen180 repeats are blue chevrons, and retrotransposons are green boxes (not to scale).

Arabidopsis centromeric repeats are transcribed and, for at least one subfamily, transcripts from one strand accumulate predominantly and are regulated posttranscriptionally by RNAi, similar to the situation in *S. pombe.* This is consistent with the tandem orientation of these large blocks of repeats: putative promoter sequences found in each repeat would be expected to align in the same orientation as each other (Figure 6). Recently, each strand of the pericentromeric repeats in *Arabidopsis* was found to differ in DNA methylation [62]. While certainly an intriguing result, these differentially methylated sequences were located outside the satellite repeats and mostly composed of retrotransposons integrated in both orientations. Therefore, the significance of this methylation for strand-specific transcription of the satellite repeats is not yet clear.

Even though they are transcribed from only one strand, tandem repeats can theoretically generate siRNA by reiterative rounds of RdRP replication and Dicer degradation [63]. The requirement for both *DCL1/AGO1* and *RDR2/DCL3* indicates both 21- and 24-nucleotide siRNAs may be involved. Although we could barely detect 21-nucleotide siRNA on blots, rare centromeric siRNA of this size has been detected by sequencing [64]. For other repeat subfamilies, both strands were silent, but transcription could be detected in mutants defective in the DNA methyltransferase MET1 and the type I histone deacetylase HDA6. These transcripts arose by read-out from LTR retrotransposons inserted among the repeats (see Figure 3E).

In fission yeast, RNAi is required to maintain normal levels of H3K9me2 at the outer pericentromeric repeats [23] and mutants have lagging chromosomes at anaphase [24,29]. A similar repeat is responsible for aspects of silencing at the mating type locus, but additional *cis*-acting elements are required for silencing in the absence of RNAi, requiring histone deacetylation instead [65]. In *Arabidopsis, dcl3* and *rdr2* mutants had little or no 24-nucleotide centromeric satellite siRNAs, yet had no detectable defects in H3K9me2 accumulation or in centromere function. This is also evidence of redundant mechanisms for maintaining H3K9me2, one RNAi based and the other depending on CG methylation and histone deacetylation of retrotransposons. However, *kyp* mutants lost most H3K9me2 without anaphase defects, although other H3K9 methyltransferases may be redundant with KYP [19], just as other Dicers are redundant with *DCL3* [48]. Nevertheless, we cannot exclude the possibility that heterochromatic association of cohesin via H3K9me2 is not required for mitotic chromosome disjunction in plants [34], which lack centrosomes and may differ from animals in this respect.

If RNAi at the centromere is dispensable but transcripts are still found, this raises the possibility that the transcripts themselves may have some function. Centromeric transcripts from CRM retrotransposons and CentC satellite repeats are associated with immunoprecipitated kinetochores in maize, although neither full-length transcripts nor siRNAs were detected on Northern blots, so that the origin and fate of these transcripts are unknown [66]. Similarly, major and minor satellite repeats are transcribed from mouse centromeres, but in this case RNA interference is thought to play a role [30,67]. Our results indicate that centromeric transcripts can be long, persist in the nucleus, and show some correlation with mitotic activity and may associate with chromosomes. Whether the transcripts serve to recruit factors in addition to the RNAi apparatus remains to be seen.

The role of DDM1 in siRNA production is unclear, but it may stabilize siRNA in a complex [39], or even promote RNA-dependent RNA polymerase, in the same way as other swi/snf helicases promote DNA-dependent RNA polymerases [68]. This could account for the difference between 106B and cen180 siRNA accumulation in *ddm1.* If siRNA processing was reduced but 106B was transcriptionally up-regulated, this could lead to an overall increase in siRNA in *ddm1.* In contrast, if cen180 repeats were not transcriptionally up-regulated, siRNA would be decreased in *ddm1.* Combined losses of siRNA and H3K9me2 are most severe in *ddm1,* and chromocenters are severely disrupted [69], indicating they may play a role in heterochromatin association even though lagging chromosomes were not observed.

### Maintenance and Reestablishment of Silencing

Silencing of centromeric repeats was restored in *met1/+* following backcrosses to WT, as was methylation [70]. However, transgene silencing and methylation were not restored in similar backcrosses, although allelic differences could be responsible [52]. Transposon methylation could also be restored in *met1/+* but only for those transposons that retained H3K9me2 and siRNA in *met1* [39]. siRNA from cen180 repeats was also retained in *met1* and *hda6,* but it was reduced in *ddm1* and F-strand silencing was not restored in *ddm1/+*. These results are consistent with a silencing complex, composed of DDM1, MET1, and HDA6, while de novo histone and DNA modification is guided by siRNA [39]. HDA6 may act downstream of MET1 as CG methylation is unaffected in *hda6* mutants [54] (Figure S4), consistent with the interaction of histone deacetylase with CG methyl-binding proteins in mammalian cells [71]. cen180 repeats transcribed in *cmt3* and *kyp* are inherited epigenetically in an active state in backcrosses, indicating silencing cannot be reestablished in trans even in the presence of cen180 siRNA, which is retained in the mutants. H3K9me2 is lost in *kyp* and may play a role in reestablishment. In *cmt3,* H3K9me2 is retained but CNG methylation is lost so both marks may be required. However, silencing of some, but not all, of these transcripts is restored in *hda6/+,* indicating that epigenetic inheritance is not simply a matter of chromosomal modification.

### Evolution of Centromeres

Centromeres are dynamic components of genome evolution. In sequenced arrays of human 171-bp satellites, the central repeats appear to be the youngest, with progressively older repeats on the outside. Formation and homogenization of new repeats are hypothesized to occur via unequal recombination, so that one repetitive sequence comes to dominate the centromere [50]. CENP-A has been proposed to co-evolve with such repeats, although binding to new, homogenous repeats has not been directly tested [72]. Transcribed repeats could also be replicated via RT and retrotransposition. New copies, being identical in sequence, would still be recognized by siRNA. As repeats age, retrotransposon insertions would recruit MET1 and DDM1, silencing the repeats transcriptionally and allowing them to diverge (Figure 6). This model predicts that younger RNAi-regulated repeats should be toward the center of the centromere and older MET1-regulated repeats should be on the flanks. In support, BLAST analysis showed that MET1-regulated repeats do indeed predominate in the sequenced portion of the genome, i.e., the exterior regions of the centromere (Figure S5). The regulation of subfamilies of centromeric repeats has diverged in the Columbia and Landsberg ecotypes, which are closely related [73]. Although the centromeric heterochromatin has not been completely sequenced in either strain, the pattern of retrotransposon insertions elsewhere in the genome is quite different [74], and this could account for the differential silencing of individual subfamilies of centromeric repeats in each strain (Figure 6). The transcription of centromeric repeats provides an attractive model for speciation. In wide crosses, paternal chromosomes might be destabilized if the pericentromeric repeats no longer match the sequence of maternal siRNA. This mechanism could contribute to hybrid incompatibility in polyploids as well as the loss of paternal chromosomes in wide crosses [75,76].

## Materials and Methods

### Plant material.

*cmt3-m5662, met1–1* (E. Richards), *ddm1–2* (E. Richards), *kyp-2* (S. Jacobsen), *hda6/sil1* (I. Furner), *dcl1–9* (S. Jacobsen), *ago1–9* mutations were introgressed into the Landsberg *erecta* ecotype. *dcl3–1* (J. Carrington) and *rdr2–1* (J. Carrington) are in the Columbia ecotype and were obtained from the Syngenta Arabidopsis Insertion Library (SAIL) collection of T-DNA insertions (Syngenta Biotechnology Inc. [SBI], Research Triangle Park, North Carolina, United States). Plants were grown 28 days (16 h light, 8 h dark) for RNA and chromatin preparation. Sources for seed stocks are indicated in parentheses.

### Transcript analysis.

RNA was prepared, reverse transcribed into DNA, and amplified as described [39]. RT-PCRs were performed with 100 ng of RNA per reaction using the OneStep kit from Qiagen (Valencia, California, United States) according to the manufacturer's protocol. Negative controls to detect contaminating DNA were performed on the RNA preparations using cen180 F + R or cen180 Fc + Rc primers but no RT. The following reaction conditions were used in each cycle: 94 °C for 20 s, 60 °C for 30 s, and 72 °C for 1 min. Southern hybridization was performed using standard methods [77]. Primer sequences were selected from conserved and variable regions of cen180 repeats, also known as pAL1 and AtCon (see Supplementary Information). The cen180 F (forward) primer is conserved in *Arabidopsis* and related species, while the R (reverse) primer is specific to subsets of repeats [2,20]. The cen180 Fc and Rc primers match conserved regions within the repeat. For CLUSTALW analysis, sequences were determined for 25 to 30 cDNA clones from each mutant. A cen180 F primer with a T3 promoter and a cen180 R primer with a T7 promoter were used to amplify cen180 repeats, and these products were transcribed in vitro to prepare strand-specific probes for in situ hybridization, performed according to the protocol of Jeffrey Long (Salk Institute for Biological Studies, San Diego, California, United States) (for protocol, see http://www.its.caltech.edu/~plantlab/protocols/insitu.pdf).

### Histone H3 methylation.

Chromatin immunoprecipitation (ChIP) was performed as previously described [39,40] using conserved F primers and R primers specific to each class of repeat. Semiquantification of the cen180 repeat ChIP data was performed by comparing PCR results from three different cycle numbers (13, 15, and 17 cycles), which were then analyzed by Southern blotting. In this way, the PCR of such highly repetitive sequences was maintained in the linear phase to avoid PCR saturation. DNA samples from each genotype were then normalized to each other by amplifying dilutions of total input DNA. Semiquantitative data were then obtained by comparing amplification with each set of primer pairs within the same ChIP extraction, which served as internal controls. In this way, control primers such as actin, whose association with lysine-9 is unclear, could be avoided. In all cases, mock precipitation with no antibody yielded little or no product. In Figure S3, PCR conditions were 17 cycles for F and R primers and 28 cycles for *dcl-* and *met-*specific primers. PCR products were blotted and probed with radiolabeled cen180 repeats.

### siRNA hybridization.

Total RNA was purified, large RNAs were removed by precipitation in 5% PEG-8000/0.5 M NaCl, and 50 μg of the resulting enriched small RNA was loaded per lane in a 10% polyacrylamide/urea gel and separated by electrophoresis [39]. The gel was electroblotted and probed with in vitro transcribed, radiolabeled RNA. Imaging was performed on a PhosphorImager (Fuji, Tokyo, Japan).

## Supporting Information

Figure S1 Northern Analysis of cen180 Transcripts Total RNA (50 μg) from WT (lane 1), *met1* mutants (lane2), and *dcl1* mutants (lane 3) were separated on an agarose gel, blotted, and probed with radiolabeled transcripts corresponding to each strand (F and R) of cen180 repeats. Heterogeneous high-molecular-weight transcripts hybridize to both strands in *met1* mutants. Prominent bands cross-hybridize with rDNA (left).
(171 KB JPG)

Figure S2 CLUSTALW Analysis of Sequences of Transcribed Repeats An unrooted tree of cen180 cDNA sequences from different mutants in the Landsberg ecotype. Sequence identifiers beginning with w are from *WT;* d, from *dcl1;* a, from *ago1;* and m, from *met1.* The *met1* branches are red. Sequences found in *WT, ago1,* and *dcl1* were not found in *met1.*
(183 KB PDF)

Figure S3 Methylation of Lysine-9 (K9) and Lysine-4 (K4) of Histone H3 at Centromeric Repeats Chromatin was immunoprecipitated with anti–dimethyl K9 antibody (K9), anti–dimethyl K4 antibody (K4), or no antibody (na). Precipitated samples and total input chromatin (tot) were then PCR-amplified with cen180 F + R primers, F + dcl–specific primers, F + met–specific primers, and 106B F + R primers.
(1.9 MB JPG)

Figure S4 Centromeric Repeat Methylation Genomic DNAs were prepared from 3-week-old plants of the indicated genotypes. Methylation changes at (A) cen180 repeats and (B) 106B repeats were assayed by digestion with HpaII (left) and MspI (right) for each repeat followed by blotting and Southern hybridization.
(775 KB JPG)

Figure S5 Comparison of cen180 cDNAs from WT (wt), *dcl3* Mutants, and *met1* Mutants with the Sequenced Region of the *Arabidopsis* Genome Sequences of 32 cDNAs from WT and each mutant in the Columbia ecotype were BLASTed against the Columbia genome (TIGR version 5, http://www.tigr.org) using a cutoff of E < 0.0001. The matches for each repeat were collected and sorted into bins according to score. Each bin is 10 score points.
(1.2 MB JPG)

Text S1 Primer Sequences The same oligonucleotide primers were used for RT-PCR, ChIP, and probe amplification.
(24 KB DOC)

### Accession Numbers

The dBEST accession numbers in GenBank (http://www.ncbi.nlm.nih.gov/Genbank) for the centromeric satellite cDNA sequences are DV671393 to DV671628.

## Abbreviations

bp: base pair
RNAi: RNA interference
siRNA: small interfering RNA
WT: wild-type
