# Supplementary material for: Differential Regulation of Strand-Specific Transcripts from Arabidopsis Centromeric Satellite Repeats
Source: PLoS Genet. 2005 Dec 23;1(6):e79. doi: 10.1371/journal.pgen.0010079 (PMC1317654; doi:10.1371/journal.pgen.0010079)
Supplement: Supplementary file 1 [file pgen.0010079.sg002.pdf]

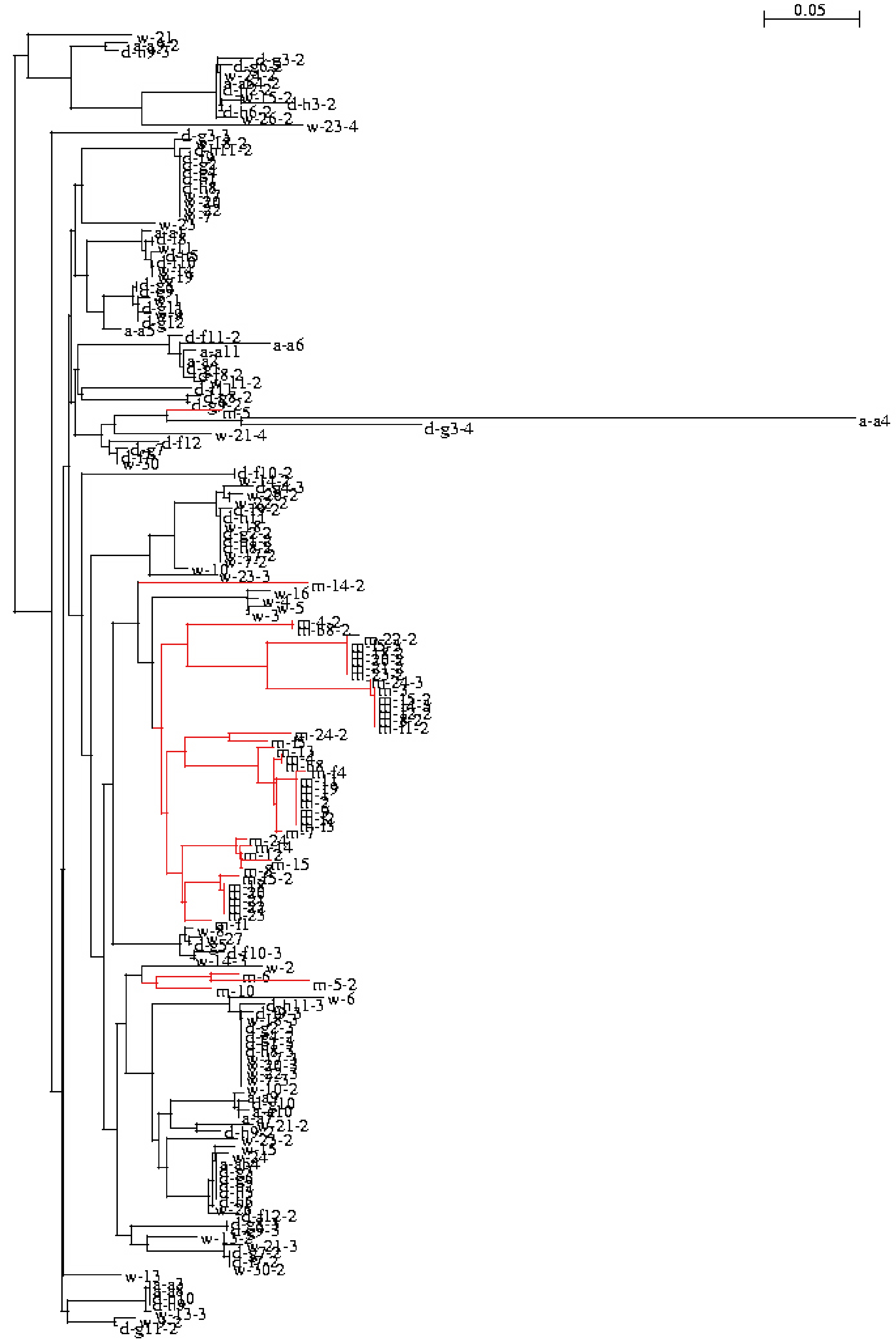
0.05
w-23-4
d-h3-2
a-a6
a-a4
d-g3-4
w-21-4
d-f12
m-14-2
m-24-2
m-15
m-5-2
m-10
w-6
w-2
d-h11-3
w-21-2
w-23-2
d-f12-2
w-21-3
w-30-2
w-13-3
d-g11-2
