# Supplementary material for: Differential Regulation of Strand-Specific Transcripts from Arabidopsis Centromeric Satellite Repeats
Source: PLoS Genet. 2005 Dec 23;1(6):e79. doi: 10.1371/journal.pgen.0010079 (PMC1317654; doi:10.1371/journal.pgen.0010079)
Supplement: Supplementary file 2 [file pgen.0010079.st001.doc]

**Supplementary Information.**

**Primer sequences.** The same oligonucleotide primers were used for RT-PCR, ChIP and probe amplification. Sequences were as follows:

cen180 F: ACCATCAAAGCCTTGAGAAGCA

cen180 R: CCGTATGAGTCTTTGTCTTTGTATCTTCT

cen180 Fc: GGTTAGTGTTTTGGAGTCGAATATG

cen180 Rc: TTGCTTCTCAAAGATTTCATGGT

106B F: TTGATTGATAGATCCCTTCTGGA

106B R: CGAGGATGGGGTAATTGAGT

106B F out: CGAAACTACTCAATTACCTCATCC

106B R out: TCCAGAAGGGATCTATCAATCAA

cen180 dcl probe (Landsberg): TTAGGCTTTTAAGAGCGCGT

cen180 met probe (Landsberg): TTTAGCTTTTGGGAACTGGT

cen180 dcl probe (Columbia): TTAGGCTTATAAGATGCAGTT

cen180 met probe (Columbia): TTACGCGTATAAGATCCGATT
